# Japan: achieving UHC by regulating payment

**DOI:** 10.1186/s12992-019-0524-4

**Published:** 2019-11-28

**Authors:** Naoki Ikegami

**Affiliations:** School of Public Health, St. Luke’s International University, Tokyo, Japan

**Keywords:** Universal health coverage, Extra billing, Balance billing, Fee schedule, Japan

## Abstract

The triple goals of Universal Health Coverage (UHC) are to cover the whole population, to reduce patients’ costs, and to expand coverage to all effective services, equitably available to all. This paper analyses the experience of Japan in achieving these goals, focusing on the central role played by the payment system. The payment system, or fee schedule, sets the price of services and pharmaceuticals, as well as the conditions that providers must comply with in order to receive payment. The fee schedule was first introduced following the enactment of social health insurance (SHI) in 1922. Initially, the SHI program covered only manual workers, who comprised a mere 3% of the population. However, the fee schedule of the largest SHI plan was subsequently adopted by all other SHI plans. From 1958, there has been only one fee schedule. Population coverage was achieved in 1961 by mandating all residing in Japan to enroll in SHI, thereby making everyone entitled to all the services and pharmaceuticals listed in the fee schedule. Next, co-insurance was capped to an affordable level by the introduction of catastrophic coverage in 1973. Lastly, extra billing and balance billing were explicitly restricted in 1984. The key to achieving and sustaining UHC goals in Japan lies in being able to contain costs and reallocate resources by revising the fee schedule.

## Background

Income inequities are reflected in various health inequities, such as life expectancy at birth [1]. Universal Health Coverage (UHC) aims to mitigate such inequalities by making health services available on the basis of the individual’s need, and not the ability to pay. UHC goals can be represented by the three dimensions of a cube [2], where the x axis concerns extending healthcare coverage to the entire population; the y axis, reducing the amount paid by the patient; and the z axis concerns expanding the services covered. The policy focus has been on the x axis, because it is the most tangible. Politicians can claim that the entire population is “covered” for “all appropriate” health services by issuing identity cards showing the bearer’s entitlement to receive health services.

There has been less attention to the y and z axes. However, unless the government explicitly defines what is actually “covered,” patients will still risk catastrophically high healthcare costs. Patients are seldom in a position to make choices as consumers, and must pay the amount demanded by the physician or hospital. In order to keep patients’ fees at an affordable level (y axis), not only should there be a limit on the co-payment (a fixed amount for a visit) and/or the co-insurance (a fixed proportion of the amount billed by the physician or hospital) for the services covered: in addition, all effective services and pharmaceuticals available at the point of delivery must be covered (z axis). Moreover, providers should be strictly regulated, to minimize extra-billing of services and pharmaceuticals not covered, and/or balance billing (more payment for services and pharmaceuticals that are covered by the public program).

The important role of the payment system in managing the y and z axes has not received adequate attention in global health policy. This commentary seeks to rectify this gap, by explaining how Japan has achieved and sustained these goals through revising the fee schedule to which all providers must adhere in order to be reimbursed by social health insurance (SHI). Although the contexts differ greatly in other countries, insights may be gained on the complexities of the issues involved in containing costs and reallocating resources.

Japan belongs to the group of countries where payment for healthcare services is strictly regulated, with an emphasis on primary care [3]. The proportion of publicly financed expenditures is the highest among the OECD countries, at 84.0% [4]. Historically, Japan’s healthcare system has been known for its high levels of health outcomes, combined with low cost and equity [5–8]. In recent years, costs have risen: the percentage of total health expenditures/GDP, which used to be lower than the average among OECD countries, has increased to 10.9%, the sixth highest, as a result of the rapidly aging demographics (the proportion of those 65 and over is 28%, the highest in the world), and the introduction of the public long-term care insurance in the context of a stagnant economy [9].

### The road to UHC in Japan

The road to UHC began in a low-key way when the Health Insurance Act was legislated in 1922 (implemented in 1927). The official goal was to maintain the health of workers so that they would contribute to the nation’s wealth. However, the actual driving force was the fear of a socialist revolution, exacerbated by the establishment of the Russian Soviet Federative Socialist Republic in 1917, and the founding of the USSR in 1922. Initially, Japan’s social health insurance (SHI) plans covered only regularly employed manual workers, a mere 3% of the population. Employers were responsible for enrolling their employees, and paid over half of the premiums. Patients did not have to pay co-insurance, because the objective was for them to return to the work force as soon as possible.

The items listed in the fee schedule determined the benefits for enrollees and the payment for providers. The fee schedule of the Government-managed Health Insurance (GMHI) for the employees of small companies set the standard because it was by far the largest SHI plan (employees of large companies had their own company-based SHI plans). The fee schedule was very simple; it had been designed by the President of the Japan Medical Association (JMA) based on the fee-for-service charges made by private practitioners, who were the JMA’s main constituents [10]. When revisions were made to the fee schedule, the JMA became the organization that represented all providers. Within the JMA, specialists did not have much power because the number of big hospitals remained few and because physicians were more concerned with their relative status within the medical profession than with being peers. At the apex were the graduates of Tokyo University. Other university-level medical schools had to have three graduates from Tokyo University on the faculty, as a condition for being established. The great majority of physicians had been “grandfathered-in” from existing practitioners, or had passed the licensing examination without having undergone formal training, or had graduated from medical schools that were not of university level.

The GMHI fee schedule set the relative value (“points”) of each item, and not the monetary amount. Pharmaceuticals were listed, because dispensing was usually performed by physicians at that time. The points were converted to monetary amounts by a conversion factor, which was calculated by dividing the GMHI premium revenue with the cumulative points billed in each prefecture. If the average number of points per enrollee was higher than the national average, the conversion factor for the prefecture would be lower. Thus, although payment was made on a fee-for-services basis, total expenditures were capped by a global budget. The conversion factor became fixed in 1943 because of wartime disruptions; since then, the government has continued to control total expenditures by setting the global revision rate (see below).

The UHC goals were achieved gradually, with different milestones for each axis. Progress on the x axis, population coverage, was achieved by gradually expanding employment-based SHI plans to other employees and their dependents in the 1930s, by introducing community-based SHI plans in 1938 for farmers and informal workers, by mandating all municipalities to establish a community-based SHI plan and by mandating all those residing in a given municipality (including non-Japanese) who were not enrolled in an employment-based plan to enroll in the community-based plan in 1958. The last measure led to virtually everyone being enrolled in a SHI plan by 1961.

Progress made on the y and z axes reflected the expansion of the population covered by SHI. On the y axis, however, there was still risk of impoverishment because, except for those formally employed, patients had to pay 50% co-insurance. This problem was mitigated in 1973 by setting a ceiling on the maximum amount of co-insurance that patients had to pay (“catastrophic coverage”). Further advances were made in 1984 when explicit rules on extra billing (charging for services not covered by the SHI) and balance billing (charging more for the services covered) were implemented.

**Fig. 1:**
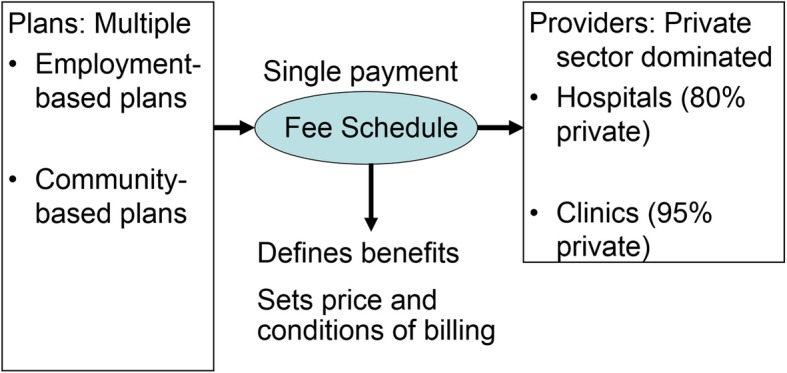
Japan’s Fee Schedule

The z axis advanced when the GMHI fee schedule was adopted by all other employment-based plans in 1943, and, in 1959, by all community-based plans. As Fig. 1 shows, setting the same payment system has meant that patients are to be treated in the same way irrespective of the SHI plan in which they are enrolled: the services covered are to be the same; likewise, the fees paid to providers are to be the same amount for the same services delivered. For the government, this meant that it could control the total flow of money from all SHI plans to all providers by revising the fee schedule. To this we now turn.

The progress mentioned above was made possible because, before and during World War II, a warfare state had to be built, with “healthy people, healthy soldiers” as the goal. After the war and the post-war chaos, there was consensus on building a welfare state, with the x axis—population coverage—as the goal. This was made possible by the rapid growth in the economy. Then, as growth began to decline from 1973, after the sharp increase in oil prices, the government became more assertive in fee-schedule negotiations with the JMA. Fees were increased less, sometimes even decreased. The power of the JMA was also beginning to decline because the relative share of its key constituents, the physicians practicing in clinics, was decreasing with the increase in hospital-based specialists. The charismatic president of the JMA for 25 years, Taro Takemi, resigned in 1983. However, despite the decline, the JMA has remained the most powerful provider group, working to mitigate cost-containment pressures on clinic-based physicians. It has maintained strong links with the Liberal Democratic Party, which has been in power almost continuously since its establishment in 1955 following the merger of the Liberal Party and the Democratic Party.

### The structure of the fee schedule

The fee schedule has played a critical role in achieving the UHC goals. As Fig. 1 shows, the fee schedule links the benefits set by the multiple SHI plans with the services delivered by the providers. The fee schedule ensures that all patients are to have the same benefits and be treated equally, because providers will be paid the same amount for delivering the same item. On the provider side, the same amount is paid for delivering the same service item, regardless of whether the provider is in the public or the private sector, and regardless of its size or geographical location. This uniform structure has facilitated the work of the government in controlling the costs and the contents of the services delivered.

Although paying the same amount for the same service item may seem unfair to big hospitals in big cities, it has been balanced by the fact that their physicians are willing to work at lower wages in Japan: average wages in large-city public-sector hospitals are 20% lower than in rural hospitals. This lower level has balanced the higher average wages paid to nurses in big-city hospitals to compensate them for the higher costs of living [11]. Paying the same amount for the same service has also helped to place the public and private sectors on an equal footing. As of 2017, the private sector had four-fifth the total number of hospitals in Japan [12]. The dominant role of the private sector has made it possible to respond to changes in priorities, while the strict fee-schedule regulations have contributed to maintaining equity.

There are now about 4000 service items and 17,000 pharmaceuticals listed in the fee schedule. Each service item is precisely defined. For example, the “first consultation visit” concerns a visit that takes places at least 30 days after the previous visit, and has been made without the physician telling the patient when to make the next visit. All other visits are “repeat consultation visits.” The fee for the former is about four times that of the latter, because it requires much more time for the physician. In addition, the fee schedule specifies the conditions of billing so as to meet quality standards and so that services will be restricted to those patients who would benefit. For example, rehabilitation therapy may be billed only by hospitals that employ the required number of therapy staff and only for patients who have suffered the injury or stroke within the past 150 days. These conditions have effectively regulated the volume of each item. Thus, although payment is made on a fee-for-service basis, there is de facto control of the volume at the level of each item.

Adherence to the conditions of billing is inspected at two levels, the first at the clearing houses that process the claims. After electronic screening, reputable physicians practicing in the local community cross-check the items billed with the patient’s diagnosis, and deny payment for the items that they judge to have been inappropriately billed. The second level involves the on-site audits that are conducted by the regional offices of the Health Ministry. Claims are cross-checked with the medical records; if there is no evidence to show that the patient had met the conditions for billing, the auditing team will order the provider to return the amount(s) inappropriately billed in the past six to twelve months. Furthermore, if the hospital and the physician are found to have deliberately forged claims, they may lose their license to deliver SHI services, which would effectively mean not being allowed to provide services in Japan.

### Revisions of the fee schedule

The fee schedule is revised every two years in order to set the global budget for total SHI expenditures, to contain pharmaceutical expenditures, and to contain or to expand the share of each item listed by increasing or decreasing its fee and/or tightening or loosening the billing conditions. The revision process is as follows.

The first step is made by the prime minister, who sets the global revision rate. Here the cumulative effects of the increases in expenditures due to expansions in service volume resulting from population aging, and from the shifts to higher-priced items because of advances in technology and so forth, are first calculated. Historically, these factors have increased health expenditures by about 2% each year. Thus, if the prime minister were to set the global revision rate at − 4%, total expenditures would remain below the current level for the next two years. However, a decrease of this magnitude would encounter vigorous opposition from the JMA and other provider groups.

After weighing all factors, the prime minister must decide. Although the estimated amounts cannot be 100% accurate, they suffice for budgeting purposes. For example, in the 2016 revision, the global revision rate was set at − 1.45%, which should have increased expenditures by 0.55% in 2017 if expenditures were to increase by 2%, as in the past. However, health expenditures decreased by 0.5% in 2016, because the actual increase from aging and advances in technology was only 0.95% [13].

The second step is revising the fee-schedule price of pharmaceuticals. Prices here have generally decreased, in the following way. For established products, prices are reduced based on a government survey of the wholesalers’ and health providers’ books on the market price and the volume of each product. Market prices are almost always found to be lower than the fee-schedule prices because pharmacies and hospitals are able to negotiate discounts from wholesalers. The price of each product has been revised so that it will be 2% higher than the average market price weighted by its volume. This process has not only led to a downward price spiral, but has also persuaded most hospitals and clinics not to dispense pharmaceuticals. The government has promoted this move by setting a higher fee if the prescription is not dispensed in the pharmacy of the clinic or hospital. The percentage of outpatients’ prescriptions dispensed by outside pharmacies has increased to 72% [14].

The price of a new product is reduced if its sales prove to be higher than predicted by the manufacturer. The government’s rationale is that the manufacturer would then be able to recover its R&D investment from the increase. Parenthetically, although cost-effectiveness analysis was formerly introduced in 2019, the results are used as additional data, to complement those on efficacy and innovativeness, in setting the launch price. The results will not be used to decide whether the product will be covered or not [15].

**Fig. 2:**
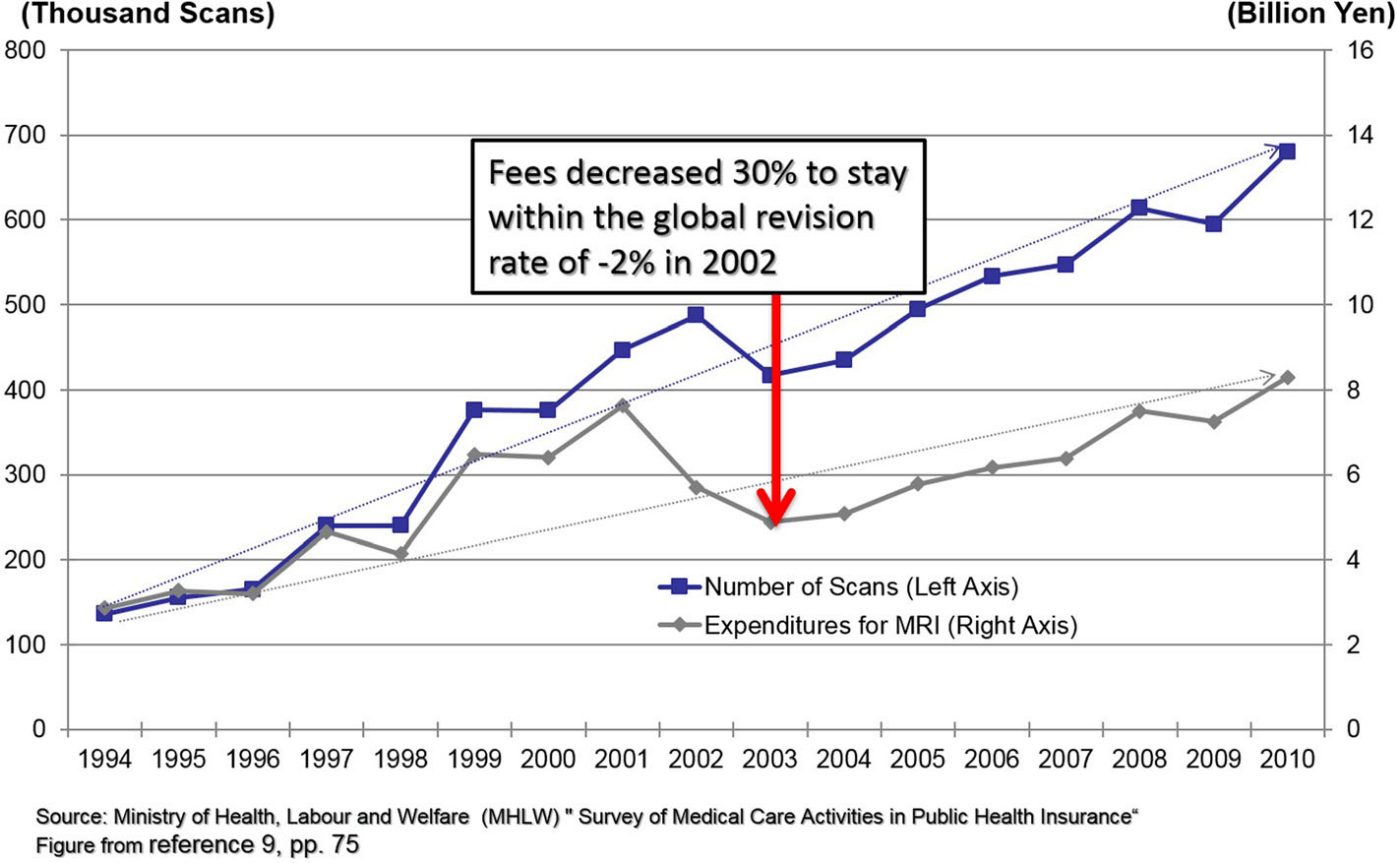
Increases in volume and costs of MRI

The third step involves revising the fee and the conditions of billing of each item within the budget set by the global revision rate and the additional savings achieved by decreasing pharmaceutical prices. The impact of revising the fee of each item is calculated from its volume in the National Claims Database. In general, if the volume of an item has been expanding inappropriately, its fee will be reduced. Figure 2 shows the effect of reducing MRI imaging fees by 30% in the 2002 revision. Although volumes have continued to increase, the cost curve has been bent, which has contributed to keeping expenditures within the global budget and allowed for increases in the fees of other items.

When a new item is listed in the Fee Schedule, its fee will be set relative to the nearest existing procedure, and not on the basis of its cost. For example, when MRI was listed in 1984, the fee was set at twice the amount for a CT scan, which did not reflect the tenfold difference in the price for purchasing the equipment at that time [16]. In the 2018 revision, surgical procedures by probe laser were listed, but the fee for a prostatectomy was essentially the same as for the conventional method. Each hospital must decide whether the loss incurred from using the laser probe would be balanced by attracting more patients and physicians. With more competition among the manufacturers, the purchase price for probe lasers is likely to be lower in the future.

Item-by-item revisions are made in order to pursue policy goals and to maintain the relative share of each sector. In both aspects, primary-care physicians in clinics have been favored because, aside from the power of the JMA, they are best positioned to meet the needs of Japan’s aging society, and have historically comprised a large share. For example, fees for physicians to make home visits to provide end-of-life care in community settings have been introduced and expanded. As a result, private practitioners in primary-care clinics who are paid on a fee-for-service basis continue to have higher incomes than specialists in tertiary hospitals, who have fixed salaries based on seniority [17].

### Extra billing and balance billing

The basis for regulating extra billing and balance billing was set out in the Health Insurance Act of 1922, which stipulated that benefits were to be in kind—in the form of services, not in cash to reimburse costs. As part of their contract, providers had to deliver all services needed by the patients enrolled in the SHI. If providers billed items that were not listed (extra-bill) or charged more than the fees set in the fee schedule (balance-bill), their contract with the SHI would be annulled. Then the provider would not be able to bill SHI, so the patient would have to bear all costs, including services covered by the SHI. (If the benefits had been in the form of cash, as with indemnity insurance, the provider would have been able to bill the patient for the balance.)

The benefit-in-kind principle was retained even after dependents of employment-based plans and those enrolled in community-based plans were enrolled and were paying 50% co-insurance. However, it was not clear whether the amount they paid was the co-insurance, or the extra-billed or balanced-billed amount. To rectify this situation, the Health Insurance Act was revised in 1984, explicitly defining and limiting the services that providers would be allowed to extra–bill or balance-bill. Extra-billing is permitted during the time a new procedure is being tested for efficacy and safety. Before conducting the test, the hospital must submit a proposal, and obtain approval from the government. While the test is being conducted, the hospital must gather data. If the evaluation results prove favorable, the procedure will be listed in the fee schedule at less than the amount that had been charged by the hospital during the test period. Heart transplant operations were listed in 2006 after following this procedure [18]. The underlying assumption has been that all procedures shown to be efficacious will be listed in the fee schedule. Balance billing is allowed mainly for hospital accommodation with better amenities, such as more floor space. However, there are restrictions on the proportion of extra-charge beds in a hospital.

Pro-market economists and industrialists have contested these strict regulations on ideological grounds [19]. However, apart from a few symbolic concessions, such as allowing a hospital to bill patients for a pharmaceutical not yet approved if the prescribing has been supervised by the research hospital conducting the test, these restrictions have basically remained unchanged. The number of patients receiving their medication in this way was only 142 as of 2018 [20]. It has proven difficult to allow greater choice while maintaining equity and ensuring quality. For this reason, the JMA has opposed deregulation, perhaps also because their main constituents are primary-care physicians in clinics, and not specialists in tertiary hospitals.

## Conclusion

The three axes of the UHC cube have been achieved using different strategies and by setting new rules. Population coverage was achieved in 1961 when it was made compulsory for all permanent residents of Japan to enroll in SHI plans (x axis). The amount of co-insurance paid by patients became capped when “catastrophic coverage” was introduced in 1973 (y axis). Explicit rules on what could be extra-billed and balance-billed were introduced in 1984 (z axis). These goals could be achieved because there has been consensus on building a welfare state and because there was rapid economic growth.

When economic growth has declined, the government has managed to contain costs by setting the global revision rate to keep expenditures within the budget. The global revision rate is not applied across-the-board: the fee and conditions of billing of each item are individually revised in order to meet budget limits and to promote or contain the delivery of each item, in line with policy goals. The JMA has been able to protect its main constituents, physicians in clinics, because of its power, and because the primary care delivered by these physicians is in line with the policy goal of addressing the needs of Japan’s aging society. Although the specifics of the design and revision of the fee schedule are unique to Japan, they do show the importance of the payment system in achieving and maintaining an equitable UHC.

## Data Availability

Not applicable.

## Abbreviations

GMHI: Government-managed Health Insurance
JMA: Japan Medical Association
MRI: Magnetic Resonance Imaging
OECD: Organisation for Economic Cooperation and Development
SHI: Social Health Insurance
UHC: Universal Health Coverage

## References

[1] Marmot MG, Smith GD, Stansfeld S (1991). Health inequalities among British civil servants: the Whitehall II study. Lancet.

[2] World Health Organization (2018). Health Financing for Universal Coverage.

[3] Reibling N, Araians M, Wendt C (2019). Worlds of healthcare: a healthcare system typology of OECD countries. Health Policy.

[4] OECD. OECD Stat 2019. https://stats.oecd.org/Index.aspx?DataSetCode=SHA. Accessed 28 July 2019.

[5] Campbell JC, Ikegami N (1988). The art of balance in health policy: maintaining Japan’s low-cost.

[6] Ikegami N (2011). Japanese universal health coverage: evolution, achievements, and challenges. Lancet.

[7] Ikegami N. Universal health coverage for inclusive and sustainable development: lessons from Japan. The World Bank, 2014. http://documents.worldbank.org/curated/en/2014/09/20278271/universal-health-coverage-inclusive-sustainable-development-lessons-japan.

[8] Ikegami N (2019). Case-study: Japan. In barber SL, Lorenzoni L, Ong P eds., Price setting and Price regulation: lessons for advancing universal health coverage*.* World Health Organization Centre for health development and OECD.

[9] Ikegami N. Financing long-term care: Lessons from Japan. Int J Health Policy Manag. 2019. 10.15171/ijhpm.2019.35. 10.15171/ijhpm.2019.35 PMC6706968 31441285

[10] Fuse S (1979). History of physicians.

[11] Ministry of Internal Affairs and Communications. Annual Report from Public Enterprises 2015. 2017.

[12] Ministry of Health, Labour and Welfare: Survey of Hospitals (2017). Ministry of health.

[13] Ministry of Health, Labour & Welfare: National Medical Expenditures 2016 Fiscal Year 2018 https://www.mhlw.go.jp/toukei/saikin/hw/k-iryohi/16/dl/data.pdf.

[14] Federation of Health Insurance Societies: Health Security Illustrated. Tokyo: Gyosei; 2018.

[15] Komoto S, Sakuramoto K, Hasegawa M (2019). Japan’s cost effective analysis evaluation – establishing cost effective analysis. Shakai hoken junpou.

[16] Hisashige A (1994). Introduction and evaluation of MRI in Japan. International J of Technology Assessment in Health Care.

[17] Ikegami N (2019). Factors determining the allocation of physicians in Japan. In barber SL, Lorenzoni L, Ong P eds, Price setting and Price regulation: lessons for advancing universal health coverage. World Health Organization Centre for health development and OECD.

[18] Japan Organ Transplant Network (2006). Heart transplants are now listed.

[19] Ikegami N (2006). Should providers be allowed to extra bill for uncovered services? Debate, resolution, and sequel in Japan. J Health Polit Policy Law.

[20] Ministry of Health, Labour and Welfare. Current state of services requested by patients not covered. Document distributed in the Working Group for Health and Long-term care of the Regulation Reform Council. https://www8.cao.go.jp/kisei-kaikaku/suishin/meeting/wg/iryou/20180403/180403iryou01.pdf. Accessed 8 July 2019.

